# Seminal Fluid Biomarkers for Early Cancer Detection: A Systematic Review

**DOI:** 10.3390/biomedicines14050966

**Published:** 2026-04-23

**Authors:** Guzel R. Sagitova, Anna V. Slizova, Andrey O. Morozov, Anastasia S. Fatyanova, Majid Ebrahimi Warkiani, Andrei V. Zvyagin, Alexey S. Rzhevskiy

**Affiliations:** 1 Institute of Molecular Theranostics, Sechenov First Moscow State Medical University, 119991 Moscow, Russia; sagitova_g_r@staff.sechenov.ru (G.R.S.);; 2 Department of Oncology, Radiotherapy and Reconstructive Surgery, Institution of Clinical Medicine, Sechenov First Moscow State Medical University, 119991 Moscow, Russia; 3 Institute for Urology and Reproductive Health, Sechenov University, 119991 Moscow, Russia; 4 School of Biomedical Engineering, University of Technology Sydney, Sydney, NSW 2007, Australia; 5 Research Center for Translational Medicine, Sirius University of Science and Technology, 354340 Sochi, Russia; 6 Australian Research Council Centre of Excellence for Nanoscale Biophotonics, Macquarie University, Sydney, NSW 2109, Australia; 7 Research Institute of Molecular and Cellular Medicine, Peoples’ Friendship University of Russia (RUDN University), 6 Miklukho-Maklaya Street, 117198 Moscow, Russia

**Keywords:** liquid biopsy, prostate cancer, testicular cancer, semen, biomarkers, cell-free DNA, microRNA, metabolomics

## Abstract

**Background:** The early detection of prostate and testicular tumors remains challenging as standard diagnostic tools often lack sensitivity and produce ambiguous results. Seminal fluid is a biologically rich medium that closely reflects the state of male reproductive tissues and has therefore emerged as a promising source of non-invasive molecular biomarkers. **Objective:** This study aimed to critically evaluate the evidence regarding cell-free DNA, RNA, proteins and metabolites in seminal fluid, and to assess their potential for improving the early detection of male reproductive cancers. **Methods:** A systematic review was performed according to PRISMA guidelines. Comprehensive searches of the PubMed and Scopus databases were conducted to identify original clinical studies analyzing molecular biomarkers in seminal fluid from patients with prostate or testicular tumors. For each study, data were extracted on biomarker types, cohort characteristics, analytical methods and diagnostic performance. **Results:** Forty-two eligible studies were included, covering multiple biomarker classes. Most were observational, single-center investigations classified as level 3b evidence. Across the different types of biomarkers, seminal fluid was associated with tumor-associated molecular changes. Alterations in the concentration, fragmentation and methylation patterns of cell-free DNA (e.g., GSTP1, RARβ2, LGALS3 and OCT3/4) distinguished malignant from benign conditions with sensitivities of up to 80–100%. RNA-based markers, including microRNAs, small non-coding RNAs, and tRNA fragments, showed improved performance in several studies, with multimarker models achieving areas under the curve (AUCs) of 0.85–0.93. Proteomic analyses identified high-specificity candidates such as TGM4, AMACR, PROS1 and DKK3. Metabolomic profiling further strengthened the diagnostic potential; reduced seminal citrate outperformed prostate-specific antigen (AUC 0.748 vs. 0.548), and reproducible shifts in amino acid and lipid profiles were observed in testicular tumors. However, substantial heterogeneity in study design, patient selection, and analytical platforms was observed. Risk of bias varied, and large prospective validation cohorts were lacking. **Conclusions:** Current evidence suggests that seminal fluid contains molecular signals associated with tumors that could be used for diagnosis. However, the available data are predominantly exploratory and methodologically heterogeneous. Before seminal fluid-based biomarkers can be considered for routine clinical implementation, robust prospective studies with standardized protocols are required.

## 1. Introduction

Cancer remains one of the leading causes of morbidity and mortality worldwide, with cancers associated with the male reproductive system, such as prostate and testicular cancer, representing significant health burdens. Prostate cancer (PCa) is the second most commonly diagnosed cancer in men, accounting for approximately 1.47 million new cases and 396,792 deaths annually [1]. It predominantly affects older men, with most cases occurring after the age of 65. Testicular cancer, while less common, is one of the most frequently diagnosed malignancies among young men aged 15 to 35 [2], with an estimated 72,031 new cases and 9056 deaths worldwide in 2022 [1]. Despite its relatively low mortality rate due to effective treatments, early detection remains crucial for preventing disease progression and improving long-term outcomes.

Current diagnostic pathways for prostate cancer usually involve prostate-specific antigen (PSA) testing, digital rectal examination, imaging (particularly multiparametric MRI) and histological verification via biopsy. However, PSA testing has well-recognized limitations, including suboptimal specificity and the risk of overdiagnosis and overtreatment [3]. Similarly, testicular cancer is primarily diagnosed through clinical examination and imaging, which may be insufficient for detecting early or subclinical disease [4]. These limitations have led to growing interest in non-invasive diagnostic approaches that can improve early detection and risk stratification.

In recent years, liquid biopsy approaches have received a great deal of attention as a means of providing non-invasive diagnoses [5]. In this context, seminal fluid has emerged as a promising diagnostic medium for malignancies of the male reproductive system. Due to its direct anatomical and functional association with the prostate, testes, and accessory glands, it contains a diverse range of molecular components, including cell-free DNA (cfDNA), circulating RNAs, tumor cells and exosomes, that reflect the molecular changes linked to tumor development [6]. These features provide a biologically plausible basis for the discovery of biomarkers.

While seminal fluid biomarkers are biologically plausible, the current evidence base is heterogeneous and largely exploratory, with limitations arising from methodological variability. The discrepancy between biological potential and clinical implementation underscores the importance of systematically mapping available data and identifying methodological limitations.

This systematic review aims to summarize the current evidence on seminal fluid biomarkers and evaluate their potential for diagnosing male reproductive cancers. It also outlines future research directions and the clinical applicability of these novel, non-invasive approaches.

## 2. Materials and Methods

### 2.1. Protocol Registration

This review was performed according to the Preferred Reporting Items for Systematic Reviews and Meta-Analyses (PRISMA) statement. The protocol was registered in PROSPERO (CRD42025649188).

### 2.2. Search Strategy

A systematic search was performed following the PRISMA guidelines (Table S1).

To identify relevant studies, a search for papers published in scientific databases (PubMed and Scopus) was performed. The search strategy combined Medical Subject Headings (MeSH) with free-text terms related to seminal fluid, liquid biopsy approaches, molecular biomarkers, and male reproductive cancers. The following search string was used in PubMed:

(“Semen”[Mesh] OR ejaculate OR “seminal fluid” OR “seminal plasma” OR sperm) AND ((“Liquid Biopsy”[Mesh] OR “liquid biopsy”) OR (“Cell-Free Nucleic Acids”[Mesh] OR “circulating cell-free DNA” OR cfDNA OR ctDNA) OR (“Neoplastic Cells, Circulating”[Mesh] OR “circulating tumor cells” OR CTC) OR (“Extracellular Vesicles”[Mesh] OR exosome*) OR (“MicroRNAs”[Mesh] OR miRNA*) OR (“RNA, Untranslated”[Mesh] OR “non-coding RNA” OR lncRNA OR mRNA) OR (“Proteins”[Mesh] OR protein*)) AND ((“Prostatic Neoplasms”[Mesh] OR “prostate cancer”) OR (“Testicular Neoplasms”[Mesh] OR “testicular cancer”)).

For Scopus, searches were performed using the TITLE-ABS-KEY fields: TITLE-ABS-KEY (“semen” OR “ejaculate” OR “seminal fluid” OR “seminal plasma” OR “sperm”) AND (“liquid biopsy” OR “circulating cell-free DNA” OR “cfDNA” OR “ctDNA” OR “circulating tumor DNA” OR “circulating tumor cells” OR “CTC” OR “exosome” OR “extracellular vesicles” OR “microRNA” OR “miRNA” OR “non-coding RNA” OR “mRNA” OR “protein”) AND (“prostate cancer” OR “testicular cancer” OR “prostatic neoplasms” OR “testicular neoplasms” OR “male cancer”).

### 2.3. Search Eligibility Criteria

We included original articles written in English that reported empirical data on seminal fluid biomarkers related to the diagnosis or prognosis of sex-specific cancers in men, specifically prostate and testicular cancer.

The exclusion criteria were as follows: review articles; editorials; commentaries; letters to the editor; meta-analyses; protocol papers; studies not involving seminal fluid as a biospecimen; non-human or in vitro studies; and articles focusing solely on fertility biomarkers in cancer survivors.

### 2.4. Study Selection and Data Extraction

Study selection was performed using the web-based Rayyan review platform. Two independent reviewers (G.S. and A.S.) screened the titles and abstracts to determine their relevance. In cases of disagreement or uncertainty, the article was included in the full-text review stage. The final selection was verified by a third reviewer (A.R.).

The same two reviewers extracted the data independently using a pre-designed Excel spreadsheet. Any discrepancies in interpretation were resolved through discussion with the third reviewer. For each study, we collected the following information: type of biomarker; the number of patients and controls; cancer type; biomarker isolation and detection methods; and reported diagnostic or prognostic utility.

### 2.5. Risk of Bias and Quality Assessment

The methodological quality and level of evidence of the included studies were evaluated using the Oxford Centre for Evidence-Based Medicine (OCEBM) Levels of Evidence framework. The risk of bias in each study was evaluated using the Quality Assessment of Diagnostic Accuracy Studies-2 (QUADAS-2) tool, which considers four key areas: patient selection, the index test, the reference standard and flow and timing. Quality assessment was performed independently by two reviewers, with any discrepancies resolved by consensus.

### 2.6. Data Synthesis

A qualitative and descriptive synthesis of the included studies was performed. Given the substantial heterogeneity in study design, patient populations, analytical platforms, and reported outcomes, quantitative meta-analysis was not feasible.

Studies were grouped according to cancer type (prostate cancer or testicular cancer) and biomarker class, including cell-free DNA, circulating RNAs, proteins, metabolites, and circulating tumor cells. For each study, key characteristics and findings were summarized, including sample size, analytical methodology, and reported diagnostic or prognostic performance where available.

Data management was performed using Microsoft Excel and Zotero 7.0.32 was used to organize references.

## 3. Results

**Figure 1:**
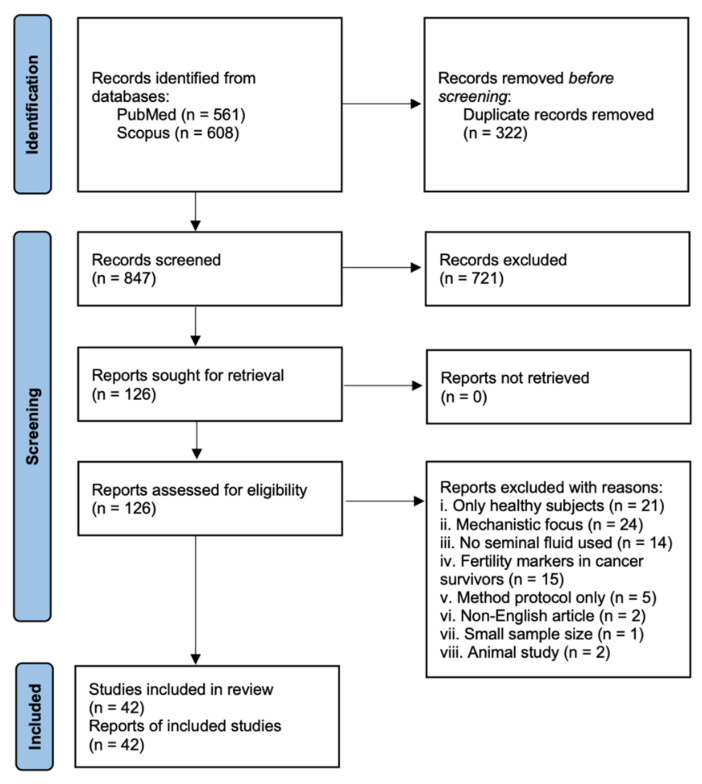
PRISMA flowchart of systematic review procedure.

A total of 1169 records were identified through database searches: 561 from PubMed and 608 from Scopus (Figure 1). After removing 322 duplicates, 847 unique records remained for screening. Following screening of titles and abstracts, 721 studies were excluded, and 126 full-text articles were assessed for eligibility.

A total of 42 studies were included in the final systematic review (Table 1). According to QUADAS-2, most studies except five had a low or some concerns risk of bias (Figure S1). Most studies had a level of evidence of 3b, which is consistent with the exploratory and early stage of research into seminal fluid-based molecular biomarkers (Table S2). In this context, evidence is primarily derived from observational diagnostic studies and pilot investigations. While these studies do not provide definitive clinical validation, they are essential for identifying biomarkers, generating hypotheses, and assessing feasibility. Therefore, they represent the most relevant and informative evidence currently available.

### 3.1. Cell-Free DN

Cell-free DNA (cfDNA) consists of short DNA fragments that are released into body fluids through apoptosis, necrosis or active secretion [49]. In cancer patients, some cfDNA originates from tumor cells and is known as circulating tumor DNA (ctDNA). This ctDNA carries tumor-specific molecular alterations, such as point mutations, DNA methylation and fragment length variations [49]. The analysis of cfDNA has emerged as a promising non-invasive approach to detecting cancer, monitoring disease and assessing tumor burden.

Due to its anatomical proximity to the prostate and testes, seminal plasma is a potentially informative matrix for cfDNA analysis in prostate and testicular cancers. This may increase the relative contribution of tumor-derived DNA compared to that in peripheral blood. However, when interpreting cfDNA signals, the biological complexity of seminal fluid should be taken into account, given that it contains secretions from multiple organs.

Several studies have investigated the diagnostic utility of cfDNA in semen for PCa.

One of the earliest studies in this field was conducted by Gonzalgo et al. (2004) [7], who examined GSTP1 promoter methylation in prostatic secretions obtained from radical prostatectomy specimens. Using a combinatorial methylation-specific PCR approach, they detected methylation in 86% of the samples analyzed. The degree of methylation correlated with tumor extent and patient age, but not with PSA or Gleason score. These results suggested that epigenetic markers in prostate-derived fluids, including seminal plasma, could be used for non-invasive cancer detection. A decade later, Zhang et al. (2015) demonstrated that promoter methylation of the *RARβ2* gene was significantly higher in the ejaculate of PCa patients than in those with benign prostatic hyperplasia (BPH) [8]. 100% of tumor samples showed elevated methylation, as confirmed by pyrosequencing. While these findings support the feasibility of epigenetic analysis in seminal cfDNA, both studies were limited by relatively small sample sizes and single-center designs.

More recent studies have focused on quantifying cfDNA levels and assessing their physical and molecular characteristics. Ponti et al. (2018) performed fluorometric quantification and electrophoretic analysis of cfDNA isolated from the semen of patients with PCa compared to healthy subjects [9,10]. The results showed that the level of cfDNA was significantly higher in samples from patients with PCa than from healthy individuals. In addition, cfDNA from patients with PCa showed a more pronounced variation in fragmentation, i.e., the length distribution of DNA fragments was broader than that of healthy individuals. Follow-up work (Ponti et al., 2019) revealed a higher prevalence of long cfDNA fragments in PCa patients compared to those with BPH or in the healthy control group [11]. This suggests that cfDNA integrity may reflect the underlying tumor biology. While these observations indicate potential diagnostic relevance, the reproducibility of these results across independent cohorts remains uncertain. Expanding the scope of molecular profiling, Škara et al. (2023) evaluated the methylation status of the *CAV1* gene in seminal plasma cfDNA [12]. Although CAV1 methylation at the CpG1 site was paradoxically higher in BPH than in PCa, it outperformed PSA in distinguishing diffuse glandular hyperplasia from cancer (area under the curve (AUC) 0.63 vs. 0.52) and further aided in differentiating indolent from potentially aggressive tumors (AUC 0.72).

Building on the search for novel epigenetic targets, Abramovic et al. (2024) assessed LGALS3 promoter methylation in seminal plasma cfDNA [13]. Their findings indicated significantly elevated methylation in PCa patients versus BPH controls, with a sensitivity of 56.4% and a specificity of 70.4%. Notably, no meaningful differences in LGALS3 methylation were observed in blood samples, and PSA levels were ineffective in discriminating between patient groups. This underscores the added diagnostic value of cfDNA analysis in semen.

The use of cfDNA as a diagnostic biomarker is still emerging in testicular cancer (TC), particularly testicular germ cell tumors (TGCTs). A study by Raos et al. (2022) examined the cfDNA methylation of OCT3/4, KITLG and MAGEC2 as potential biomarkers for testicular seminoma [14]. Their findings revealed differential DNA methylation for these genes in both blood and seminal plasma. The authors proposed two screening models: a CpG1 (MAGEC2)/CpG1 (OCT3/4) panel for seminal plasma, and a CpG8/CpG9/CpG10 panel in KITLG for blood. While the seminal fluid-based model demonstrated higher sensitivity (87% vs. 71%), the blood-based model showed higher specificity (79% vs. 60%), suggesting its potential as a more reliable tool for the early detection of testicular seminoma. An earlier study by the same group (Raos et al. 2021) analyzed copy number variants of four genes (*NANOG*, *KITLG*, *MAGEC2* and *RASSF1A*) in seminal cfDNA from seminoma patients before and after surgery, as well as in healthy controls [15]. While statistical significance was not reached, the results indicated a potential decrease in CNVs in postoperative samples compared to preoperative ones, suggesting a possible application of cfDNA in monitoring treatment response. Together, these findings support the feasibility of cfDNA analysis in seminal fluid for both detection and surveillance of male reproductive cancers.

### 3.2. Circulating RNAs in Prostate Cancer Diagnosis

Circulating RNAs, particularly microRNAs (miRNAs) and other small non-coding RNAs (sncRNAs), are increasingly recognized as valuable biomarkers for diagnosing cancer [50]. These RNA species in seminal plasma are derived from multiple cellular sources, including prostate epithelial cells, germ cells and accessory gland secretions. They are often protected from degradation by being encapsulated in extracellular vesicles or by being associated with protein complexes.

Numerous studies have examined seminal RNA profiles in an attempt to enhance the sensitivity and specificity of PCa detection beyond traditional PSA testing.

Initial work by Roberts et al. (2015) demonstrated that incorporating a multimarker panel of ejaculate-derived PCA3 and Hepsin mRNAs, selected miRNAs (e.g., miR-200c and miR-125b) and serum PSA into the diagnostic process improved diagnostic performance (AUC = 0.869) compared to PSA alone [16]. This was followed by a more focused analysis (Roberts et al., 2016) of PCA3, PSA and β2-microglobulin RNAs in ejaculate [17]. The authors found that the PCA3:β2M ratio in ejaculate showed improved discrimination between prostate cancer and non-malignant conditions compared to both serum PSA and post-ejaculation urine RNA markers, although overall diagnostic performance remained moderate (AUC = 0.717). Further expanding the landscape of RNA-based biomarkers, Ruiz-Plazas et al. (2021) introduced a TWEAK-regulated panel of exosomal oncomiRNAs [18]. Their study revealed significantly elevated levels of miR-221-3p, miR-222-3p and miR-31-5p in the semen of patients with high-risk prostate cancer, while levels of miR-193-3p and miR-423-5p were reduced in urine collected after a digital rectal examination. A composite model incorporating exo-oncomiRs 221-3p and 222-3p, as well as TWEAK, correctly classified 87.5% of cases, demonstrated improved performance compared to PSA alone, with an absolute difference of 23.6%.

Barceló et al. (2019, 2020) and Mercadal et al. (2020) conducted systematic evaluations of exosomal microRNAs (miRNAs) in semen as potential PCa biomarkers [19,20,21]. A seminal study by Barceló et al. (2019) showed that a combination of miR-142-3p, miR-142-5p and miR-223-3p in sperm exosomes with serum PSA yielded an area under the curve (AUC) of 0.911 in patients with PSA in the diagnostic ‘gray zone’ (4–10 ng/mL), reducing unnecessary biopsies by over 30% [19]. In a follow-up study, the same group examined cell-free miRNAs and proposed a new model incorporating miR-142-3p, miR-223-3p and miR-93-5p (AUC = 0.858), with further models distinguishing tumor risk grades [20]. Mercadal et al. (2020) emphasized that the method of EV isolation significantly affects the profile of detected miRNAs, although combined biomarker models showed moderate to high diagnostic performance across methods (AUC > 0.85–0.93) [21]. Abramovic et al. (2021) examined miRNAs in patients with elevated PSA levels and noted no significant differences in expression between PCa and BPH [22]. However, they reported positive correlations between several miRNAs and serum PSA levels.

In another study, Lorente et al. (2021) added another RNA subclass and conducted comprehensive RNA sequencing of seminal plasma [23]. They identified sncRNAs—particularly miR-143 and miR-199a—as being differentially expressed in prostate pathologies compared to controls. Notably, RNA profiles remained stable even in vasectomized men, indicating the broad applicability of semen-based RNA diagnostics. Their functional analysis linked these miRNAs to tumor-related pathways, such as transcription regulation and extracellular matrix remodeling.

In a significant recent development, Ferre-Giraldo et al. (2024) examined tRNA-derived fragments (tsRNAs) in seminal small extracellular vesicles (sEVs) from individuals with moderately elevated prostate-specific antigen (PSA) levels (4–18 ng/mL) [24]. Using sequencing and RT-qPCR, they identified four dysregulated 5′ tRFs, notably 5′ tRF-Glu-TTC-9-1_L30 and 5′ tRF-Val-CAC-3-1_L30, which were overexpressed in PCa patients compared to controls and BPH patients. A multivariate model combining these tsRNAs with PSA improved the accuracy of diagnosing clinically significant PCa (Gleason ≥ 7) to an AUC of 0.756. This approach shows promise in reducing overtreatment and guiding risk stratification.

Overall, these findings demonstrate that seminal RNA biomarkers—spanning mRNAs, miRNAs, sncRNAs and tsRNAs—can substantially improve PCa detection, particularly in diagnostically ambiguous cases. Models that integrate RNA profiles with PSA have demonstrated improved diagnostic performance compared to PSA alone in several studies, showing potential utility for early detection, tumor grading and supporting biopsy decisions. However, biological variability and methodological inconsistency, particularly with regard to EV isolation and RNA quantification, continue to pose significant challenges to standardization and clinical translation. Future large-scale validation studies are essential to confirm these promising early results.

### 3.3. Circulating RNAs in Testicular Cancer Diagnosis

Several studies have investigated the potential of sncRNAs circulating in seminal fluid as biomarkers for testicular germ cell tumors and their precursor, germ cell neoplasia in situ (GCNIS). The expression of sncRNAs reflects molecular alterations in testicular tissue and may be influenced by spermatogenic activity.

Pelloni et al. (2017) found that certain microRNAs (miRNAs) that were found to be upregulated in serum, such as miR-371 and miR-372, were instead found to be downregulated in the seminal plasma of TGCT patients [25]. This suggests that there are tissue-specific regulatory mechanisms involved in the release of miRNAs. The researchers also observed significantly higher levels of miR-142-3p (~5-fold increase, *p* < 0.001) and lower levels of miR-34b (~8-fold decrease, *p* < 0.001) in seminal plasma, suggesting their potential as diagnostic indicators. Radtke et al. (2019) examined miR-371a-3p, one of the most widely studied TGCT biomarkers in blood, and found no significant differential expression in seminal plasma between TGCT patients and controls [26]. However, its levels correlated strongly with sperm count and concentration, suggesting that spermatozoa are a major source of this RNA. Spiller et al. (2020) confirmed this, finding that levels of both miR-371a-3p and CRIPTO were consistently higher in seminal plasma than in serum [27]. Interestingly, CRIPTO was detectable in 92% of azoospermic men, suggesting that it originates from testicular tissue beyond sperm cells. Although their small TGCT subgroup (n = 4) showed a trend towards increased CRIPTO levels, the small sample size limited the possibility of making a definitive interpretation.

Mørup et al. (2021) conducted further evaluation of seminal sncRNAs in patients with TGCT and GCNIS, identifying hsa-mir-6782-5p as significantly reduced (2.3-fold, *p* = 0.02) in TGCT [28]. Despite notable biological variability driven by factors such as the presence of GCNIS and the degree of spermatogenesis, this study confirmed the feasibility of small RNA-based semen testing. Dupont et al. (2023) used high-throughput sequencing to compare the sncRNA profiles of semen in TGCT patients and healthy men [29]. While no significant global differences were observed, stratifying patients by semen quality revealed clear molecular separation: 229 differentially expressed sncRNAs were identified in TGCT patients with poor sperm quality versus 11 in patients with normal parameters. The most promising candidates were found to be miR-224, piR-has-23921 and mature-tRNA-His-GTG, which demonstrates the importance of accounting for semen quality when conducting TGCT RNA studies for diagnostic purposes.

Building on these findings, Ferrara et al. (2024) investigated miRNA expression in seminal plasma from a uniquely high-risk cohort: infertile men, a population with an elevated incidence of TGCT [30]. By comparing TGCT patients with both fertile and subfertile controls, they identified consistent downregulation of miR-221-3p and miR-222-3p, as well as upregulation of miR-126-3p, in TGCT cases. ROC curve analysis revealed strong diagnostic performance for miR-126-3p (AUC = 0.833), highlighting its potential to distinguish TGCT even within clinically ambiguous, fertility-compromised populations. Pathway analysis linked the identified miRNAs to cancer-relevant signaling cascades, including the prolactin and FoxO pathways, further supporting their biological relevance. However, these findings are limited in their generalizability due to the small sample size and selected population.

Overall, the characterization of circulating RNA biomarkers in seminal fluid for testicular cancer remains insufficient. Although several candidate molecules have been identified, the results are inconsistent and are greatly affected by biological and pre-analytical factors, particularly sperm quality and spermatogenic activity. The limited number of studies, small sample sizes and lack of standardized methodologies further restrict the interpretability of the available data. Consequently, the current evidence base should be considered exploratory and substantial additional research is required to clarify the potential clinical role of seminal RNA biomarkers in testicular cancer.

### 3.4. Circulating Tumor Cells

Circulating tumor cells (CTCs) are cancerous cells that have detached from primary or metastatic tumors and entered the bloodstream or other bodily fluids, such as seminal fluid (SF) [51]. These cells play a critical role in cancer progression, as they are implicated in the dissemination of cancer to distant sites, a process known as metastasis. Although most studies have focused on the detection of CTCs in blood, seminal fluid has been suggested as an alternative matrix for prostate cancer due to its proximity to the primary tumor site.

Recent studies have explored the potential of CTCs in SF as a biomarker for prostate cancer diagnosis. Rzhevskiy et al. (2022) investigated the use of a novel microfluidic device to isolate CTCs from SF in prostate cancer patients and assessed its correlation with established prognostic markers [31]. They found a weak correlation between CTC count and PSA levels (r = 0.40), but a moderate correlation with Gleason score (r = 0.63 per mL of SF; r = 0.73 for total CTC count), suggesting a potential prognostic role for CTC enumeration. Notably, CTCs were detected in all PCa patients (n = 15) with significantly higher numbers compared to previous blood- and urine-based methods, suggesting that SF may be a more effective medium for liquid biopsy in PCa.

In contrast, Saitta et al. (2023) investigated the feasibility of semen-based liquid biopsy for PCa by assessing patient compliance with self-sampling and isolating CTCs using multi-color flow cytometry [32]. They found that only 18.2% of invited patients successfully provided a SF sample, with younger age and lower prostate volume being significant predictors of collection success. While prostate-derived cells were more abundant in SF than urine, the fraction of putative CTCs (PSMA + EpCAMhigh) remained low (~2%) and did not correlate with clinical parameters such as Gleason Score or TNM stage.

Overall, these findings indicate that, while CTC detection in seminal fluid is technically feasible, its clinical usefulness is unclear. Limitations include low patient compliance, methodological variability and an absence of consistent associations with clinical outcomes.

### 3.5. Protein Biomarkers

Seminal plasma is a rich proteomic environment that reflects prostate and testicular physiology, making it a promising yet underutilized source of protein biomarkers for the early detection of cancer. Several studies have examined seminal proteins in order to identify indicators that could be used for the diagnosis and prognosis of PCa and TGCTs. These studies have employed a variety of technologies, including immunoassays, mass spectrometry and immunocytochemistry.

One of the earliest studies in this field was conducted by Tajiri et al. (2008), who analyzed the glycosylation patterns of PSA in seminal plasma and serum [33]. They discovered that PSA isoforms associated with cancer exhibited distinct fucosylation and sialylation profiles, particularly an increase in α2,3-linked sialic acids in PSA from PCa patients’ serum compared to the α2,6-linked forms found in PSA from the seminal plasma of healthy individuals. These structural changes suggested that glycoform-specific PSA analysis could improve diagnostic specificity. Around the same time, van Casteren et al. (2008) investigated the non-invasive detection of OCT3/4-positive cells in the semen of men at high risk of cancer [34]. This marker was present in 100% of patients with carcinoma in situ (CIS) and in 75% of TGCT patients with adjacent CIS. However, it was absent in normospermic controls. This makes OCT3/4 a highly specific indicator of early testicular neoplasia.

Zenzmaier et al. (2011) investigated levels of the Dickkopf-related protein 3 (Dkk-3) in the seminal plasma of men undergoing prostate biopsies [35]. Dkk-3 concentrations were significantly higher in patients with PCa, and, although its standalone diagnostic performance was moderate (AUC = 0.633), combining it with serum PSA improved accuracy to 0.710 and to 0.777 in long-term follow-up cohorts. However, Nielsen et al. (2011) raised important concerns about the specificity of semen-based diagnostics, demonstrating that TGCT markers such as AP-2γ and OCT3/4 are also expressed in benign urogenital epithelial cells [36]. To mitigate false positives, they introduced a double-staining approach combining nuclear marker detection with alkaline phosphatase reactivity, thereby improving CIS specificity.

Saraon et al. (2012) identified protein S (PROS1) as a strong candidate for identifying high-grade PCa [37]. Seminal PROS1 levels were significantly higher in patients with Gleason ≥ 7 disease, with an AUC of 0.875 achieved for distinguishing aggressive cases from indolent or benign ones. Neuhaus et al. (2013) used capillary electrophoresis–mass spectrometry to analyze seminal plasma peptides, identifying 21- and 5-polypeptide panels for PCa detection and an 11-peptide signature for Gleason score stratification [38]. These included fragments of semenogelin, prostatic acid phosphatase, and stabilin-2. The authors emphasized the high pre-analytical stability of seminal plasma as a diagnostic medium.

Hoei-Hansen et al. (2007) and Satie et al. (2009) investigated TGCT biomarkers in semen, including the fetal germ cell markers AP-2γ and OCT3/4, as well as the cancer testis antigens MAGE-A4 and NY-ESO-1 [39,40]. While these markers exhibited elevated expression in patients with TGCT and CIS, their presence in certain healthy individuals underscored the necessity of multimodal diagnostic strategies. Combining marker detection with standard semen analysis improved diagnostic performance to ~90%, indicating their potential as supplementary tools rather than standalone indicators.

Lippert et al. (2015) examined C-type natriuretic peptide (CNP) and its precursor (proCNP) in seminal plasma and noted an elevation between 1.8 and 2.2-fold in PCa patients [41]. These levels correlated with tumor burden and risk of biochemical recurrence. Interestingly, however, proCNP was not elevated in blood, which reinforces the tissue specificity of seminal biomarkers. Karakosta et al. (2016) assessed all 15 kallikrein-related peptidases (KLKs) in seminal fluid using mass spectrometry and immunoassays [42]. KLK4 was detectable, but there was no significant difference between PCa and control groups, which limits its standalone utility (AUC = 0.64). Nevertheless, the study emphasized the effectiveness of multiplexed proteomic assays for biomarker validation.

Etheridge et al. (2018) evaluated the presence of alpha-methylacyl-CoA racemase (AMACR) in semen using a chemiluminescent enzyme-linked immunosorbent assay (ELISA) [43]. They found that AMACR levels were significantly higher in PCa patients than in controls. At a cut-off of 76 ng/mL, the sensitivity was found to be 85.7%, while the specificity was found to be 53.3%. Combining this with patient age improved specificity to 66.7%. Despite being affected by sample degradation, AMACR showed potential as a non-invasive marker of clinically significant PCa. Drabovich et al. (2019) took a multi-omics approach, combining selected reaction monitoring (SRM) mass spectrometry and immunoassays, to identify prostate-specific androgen-regulated proteins in seminal plasma [44]. Their most promising candidate, transglutaminase 4 (TGM4), was found to be 3.1-fold higher in PCa patients, achieving 92% specificity at a cut-off of 1.74 µg/mL and outperforming PSA. When paired with PAEP and other candidates, TGM4 showed enhanced diagnostic value, though broader validation was recommended.

Finally, Ruiz-Plazas et al. (2019) investigated a biomarker panel comprising soluble TWEAK (sTWEAK), Fn14, KLK2, CXCR2 and CCR3 mRNA from seminal cell sediment, alongside serum glucose and PSA [45]. This integrated model achieved an AUC of 0.913 and 85.7% specificity for distinguishing aggressive from indolent PCa. Decreased sTWEAK and elevated Fn14 mRNA levels were notably associated with high-risk disease, suggesting that the TWEAK/Fn14 axis may play a role in PCa progression. The study emphasized the importance of combining seminal, metabolic, and clinical parameters for improved risk stratification.

In summary, seminal plasma proteomics has revealed numerous promising biomarkers for PCa and TGCT detection, including structural proteins, enzymes, transcription factors and post-translational modifications. Although individual markers often have moderate specificity or reproducibility, integrated multimarker models—especially when combined with PSA and clinical features—demonstrate significantly enhanced diagnostic and prognostic performance. Nevertheless, pre-analytical standardization, larger validation cohorts and assay harmonization are necessary before these biomarkers can be used in routine clinical practice.

### 3.6. Metabolic Biomarkers in Seminal Fluid for Prostate and Testicular Cancer

In addition to nucleic acids and proteins, seminal plasma contains a variety of small metabolites that reflect the biochemical processes occurring in the prostate and testes. Consequently, metabolomic profiling of seminal fluid has emerged as a promising, minimally invasive approach to improving cancer detection and risk stratification, particularly in patients with inconclusive PSA test results or subclinical disease.

One of the earliest and most thoroughly characterized metabolic candidates is citrate, a key prostate-secreted metabolite. Gregório et al. (2019) investigated seminal citrate concentration in men with persistently elevated PSA, comparing 31 patients with clinically significant prostate cancer to 28 controls with BPH and negative biopsies [46]. Using high-resolution nuclear magnetic resonance (NMR) spectroscopy, they found that the median citrate concentration was significantly lower in csPCa patients (3.93 mM/L) than in the control group (15.53 mM/L), despite similar PSA levels. ROC analysis showed that [CITRATE] was more accurate for diagnosis than PSA (AUC = 0.748 vs. 0.548), suggesting that citrate depletion could be a reliable and inexpensive biomarker for prostate malignancy in cases where PSA results are ambiguous. This study supports the use of metabolic profiling alongside traditional PSA testing and has the potential to reduce unnecessary biopsies.

Oxidative stress has also been implicated in prostate carcinogenesis, prompting investigations into whether reactive oxygen species (ROS) could be used as a diagnostic marker. Barrio-Muñoz et al. evaluated ROS levels in seminal plasma from 43 men undergoing prostate biopsy [47]. Although mean ROS levels were slightly higher in patients diagnosed with PCa (0.04594 ± 0.03197) than in biopsy-negative individuals (0.04537 ± 0.03827), these differences were not statistically significant. Nevertheless, the authors emphasized that ROS quantification could be valuable when integrated with other biomarkers, and they suggested that methodological optimization could enhance its discriminatory power. Their findings reinforce the idea that no single metabolic parameter is likely to be sufficient on its own, but that multi-analyte models incorporating oxidative stress indicators could enhance diagnostic precision.

Seminal metabolomics has also shown potential beyond PCa, particularly in testicular cancer. In a pilot study, Lakpour et al. (2024) used Raman spectroscopy to compare the metabolic fingerprints of serum and seminal plasma in nine patients with TC and ten fertile controls [48]. While there were no significant differences in serum profiles, seminal plasma exhibited marked metabolic alterations. TC patients exhibited elevated levels of phenylalanine, tyrosine, lipids, proteins, and phenols (*p* < 0.001), with phenylalanine showing the most pronounced increase. These results emphasize the biochemical specificity of seminal plasma as a diagnostic tool for testicular malignancy, suggesting that metabolite-based signatures, particularly aromatic amino acids, could be used as novel biomarkers for the early detection of TGCT.

Taken together, these studies emphasize the potential of seminal fluid metabolites for diagnosis in both prostate and testicular cancers. Citrate depletion, redox imbalance, and amino acid accumulation each reflect underlying tumor-associated metabolic reprogramming. Although current evidence is based on small cohorts, the consistency of results across studies supports further investigation. Integrating metabolic data with molecular and clinical markers could lay the groundwork for future non-invasive diagnostic platforms.

## 4. Discussion

This systematic review summarizes the current evidence on seminal fluid biomarkers for detecting and characterizing prostate and testicular cancers. Across the 42 studies included in the review, seminal fluid was investigated as a source of cell-free DNA, circulating RNAs, proteins, metabolites and circulating tumor cells [7,8,9,10,11,12,13,14,15,16,17,18,19,20,21,22,23,24,25,26,27,28,29,30,31,32,33,34,35,36,37,38,39,40,41,42,43,44,45,46,47,48,50,51]. Taken together, these studies suggest that seminal fluid contains tumor-associated molecular signals that could provide valuable information in the context of male reproductive cancers. However, the available evidence remains largely exploratory, with most studies being observational, single-center and based on relatively small cohorts. Accordingly, the findings should be interpreted as proof of concept rather than as evidence of readiness for clinical implementation.

There is a strong biological rationale for studying seminal fluid. As it is anatomically and functionally connected to the prostate, testes, epididymis and accessory glands, it could offer access to molecular material that is less diluted than that found in peripheral blood [52,53]. This feature is particularly relevant in the context of prostate cancer, given that the prostate contributes substantially to seminal composition. However, this complexity also represents an important limitation. Seminal fluid is a heterogeneous mixture of secretions from multiple tissues, and the precise cellular origin of detected analytes is often unclear [6]. This has direct implications for tumor specificity, particularly when attempting to distinguish signals arising from malignant tissue from those associated with benign prostatic hyperplasia, inflammation, spermatogenic activity or normal glandular function.

Of the various biomarker classes, cfDNA, RNA and protein-based approaches have produced the most consistent results in prostate cancer studies. Studies of cfDNA have revealed distinct methylation patterns and fragmentation profiles in cancer patients compared to controls [7,8,9,10,11,12,13]. Similarly, RNA-based biomarkers, particularly miRNAs and other small non-coding RNAs, have demonstrated enhanced diagnostic performance in combination with PSA in several studies [16,17,18,19,20,21,22,23,24]. Proteomic analyses have identified candidate proteins associated with tumor presence and aggressiveness, including TGM4, AMACR and PROS1. These proteins often demonstrate enhanced performance when incorporated into multimarker panels [37,43,44]. Metabolomic studies have also suggested that alterations such as reduced citrate levels may provide additional diagnostic information in certain clinical situations [46].

However, despite these encouraging findings, the reported diagnostic performance varies considerably across studies. Differences in study design, patient selection, analytical platforms, normalization strategies and outcome definitions limit direct comparability. Furthermore, several high-performing models were developed in selected or enriched cohorts, which may overestimate their performance in broader clinical populations [18,19,20,21,24,44,45]. Therefore, claims regarding improved performance relative to PSA should be interpreted cautiously and in the context of the individual study design.

By contrast, the evidence for testicular cancer is limited and inconsistent. Although several studies have identified potential biomarkers in the form of RNA, proteins and metabolites [14,15,25,26,27,28,29,30,34,39,40,48], the number of available investigations is relatively small and many of these are exploratory or pilot studies. Interpretation is further complicated by the strong influence of semen parameters, including sperm concentration, motility and spermatogenic activity, on molecular profiles [26,27,28,29,30]. These biological confounders make it difficult to distinguish tumor-specific signals from physiological variation. Consequently, the clinical utility of seminal biomarkers in testicular cancer remains uncertain.

This review highlights a major challenge: the lack of standardization in both pre-analytical and analytical procedures. Studies differed substantially in terms of sample collection methods, timing of ejaculation, processing protocols, storage conditions and biomarker isolation techniques [9,10,11,19,20,21,29,38,42]. These factors are known to influence measurements of biomarkers, including the integrity of cfDNA, the stability of RNA, the composition of extracellular vesicles, the degradation of proteins, and metabolomic profiles. For instance, RNA-based studies have revealed that the isolation method of extracellular vesicles significantly impacts miRNA profiles [21], while testicular cancer studies demonstrate the strong dependence of RNA expression on semen quality [26,29]. Without standardized protocols, reproducibility across studies remains limited.

In addition to methodological variability, practical and translational barriers must also be considered. For instance, patient compliance with semen collection may be suboptimal in routine clinical settings, particularly among older individuals [32]. Furthermore, regulatory and logistical challenges, including assay standardization, demonstrating cost-effectiveness and integrating into existing clinical workflows, remain unresolved. These factors are critical for clinical adoption and may limit the feasibility of widespread implementation, even if diagnostic performance improves.

From a clinical perspective, seminal fluid biomarkers are more likely to complement established diagnostic approaches than replace them. In the case of prostate cancer, for example, they could be useful in refining risk stratification, particularly for patients with borderline PSA levels, or in supporting decisions about biopsies [19,20,24,44,45,46]. For testicular cancer, potential applications are less clearly defined, but they may include use in selected high-risk populations or in exploratory monitoring settings [14,15,27,30]. It is important to note that future studies should clearly define the intended clinical use cases, as performance requirements differ substantially between screening, diagnosis, and prognostic applications. Looking ahead, multimodal diagnostic strategies that integrate cfDNA, RNA, proteins and metabolites from seminal fluid—either alone or in combination with PSA—may provide a more comprehensive assessment of tumor biology. Such approaches could improve diagnostic accuracy and enable more refined risk stratification. Advanced analytical methods, including machine learning-based models, could enhance the interpretation of multidimensional biomarker data further; however, their performance would depend on the availability of large, well-annotated and standardized datasets.

The overall methodological quality of the included studies reflects the field’s early stage. While most studies showed a low or moderate risk of bias according to QUADAS-2, the level of evidence was predominantly 3b and the reporting was often inconsistent. This highlights the need for more rigorous study design, adherence to reporting standards and transparent documentation of analytical workflows.

## 5. Conclusions

Seminal fluid is a promising non-invasive source of biomarkers for the early detection and risk stratification of prostate and testicular cancers. Studies across molecular classes, including cell-free DNA (cfDNA), RNA, proteins and metabolites, have demonstrated the potential to complement PSA-based diagnostics, particularly when integrated into multimarker models. Despite these encouraging results, the clinical translation of these findings is limited by methodological variability and small patient cohorts. Standardized protocols and large-scale validation are essential to establish seminal fluid biomarkers as a routine diagnostic tool for male cancers.

## Figures and Tables

**Table 1:** Overview of Seminal Biomarkers Evaluated for Prostate and Testicular Cancer Diagnosis.

Authors, Year	Patient Type	Number	Marker Type	Marker	Isolation Method	Detection Method	Performance
Cell-Free DNA							
Gonzalgo, M.L. et al., 2004 [7]	Clinically localized prostate adenocarcinoma	100	DNA methylation promotor	GSTP1 promoter	Prostatic secretions were obtained by gently squeezing the prostatectomy specimens after surgical extirpation	Bisulfite conversion of DNA. MSP—Methylation-specific PCR	Diagnostic
Zhang, T., et al., 2015 [8]	PCa/BPH	43/40	DNA methylation	RARβ2 promoter methylation	DNeasy Blood & Tissue Kit	Pyrosequencing	Diagnostic
Ponti et al., 2018 [9]	PCa/healthy controls	6/3	cfDNA	Concentration and fragmentation of cfDNA	QIAamp Circulating Nucleic Acid Kit	fluorometric assay, electrophoresis	Diagnostic
Ponti et al., 2018 [10]	PCa/BPH/healthy controls	18/25/13	cfDNA	Concentration of cfDNA	QIAamp Circulating Nucleic Acid Kit	Qubit ssDNA Assay Kit	Diagnostic
Ponti et al., 2019 [11]	PCa/BPH/healthy controls	30/33/21	cfDNA	Concentration and fragmentation of cfDNA	QIAamp Circulating Nucleic Acid Kit	Qubit ssDNA Assay Kit, electrophoresis	Diagnostic
Škara et al., 2023 [12]	PCa/BPH	40/40	cfDNA	Methylation of CAV1	NucleoSnap cfDNA kit and Nucleospin	qPCR, bisulfite conversion, pyrosequencing	
Abramovic et al., 2024 [13]	PCa/BPH	42/55	cfDNA methylation	LGALS3 methylation in cfDNA	Commercial kit Macherey-Nagel’s NucleoSnap or NucleoSpin	Pyrosequencing	Diagnostic
Raos et al., 2022 [14]	Seminoma/healthy controls	24/37	cfDNA	OCT3/4, KITLG and MAGEC2 methylation	NucleoSnap cfDNA kit and vacuum pump (Qiagen)	Quant-iTTM PicoGreen^®^ dsDNA detection kit, spectrofluorometer, bisulfite conversion and pyrosequencing	Diagnostic
Raos et al., 2021 [15]	Seminoma/healthy controls	24/35	cfDNA	Copy number variants of NANOG, KITLG, MAGEC2, and RASSF1A	NucleoSnap cfDNA kit and vacuum pump (Qiagen)	Quant-iTTM PicoGreen^®^ dsDNA detection kit + spectrofluorometer; ddPCR	Diagnostic
**Circulating RNAs**							
Roberts, M.J. et al., 2015 [16]	Men suspected of having PCa (confirmed/disproved by subsequent biopsy) 66—mRNA 20—miRNA	152 (98/54) +18 patients (12%) demonstrated PCa on further biopsies	mRNA, miRNA	mRNA—PCA3 and Hepsin; miRNA—miR-200c, miR-375, miR-200b, miR-125b	Percoll, TRIzol-based RNA extraction, RNeasy kit	mRNA: RT-qPCR; miRNA: sRNA-seq	Diagnostic
Roberts, M.J. et al., 2016 [17]	PCa/nonPCa	Comparison cohort: 25/13 Extended PEUW cohort: 36/26	mRNA, lncRNA	PSA, β2-microglobulin and PCA3	Centrifugation (Percoll), TRIzol-based RNA extraction	RT-qPCR	Diagnostic
Ruiz-Plazas et al., 2021 [18]	patients with high-risk (ISUP Group III, IV and V) and low-risk (ISUP Group I and II) PCa	40/57	miRNA + protein	exo-oncomiR-221-3p, exo-oncomiR-222-3p, exo-oncomiR-31-5p, TWEAK	exoRNeasy Serum/Plasma Maxi Kit or Midi Kit (Qiagen)	RT-PCR	Prognostic
Barceló et al., 2019 [19]	PCa–vasectomized/PCa–non-vasectomized/BPH/healthy controls	8/16/7/8	Exosomal miRNA	miRNA panel (diagnostic: miR-142-3p, miR-142-5p, miR-223-3p; prognostic: miR-342-3p, miR-374b-5p)	Filtration and ultracentrifugation-based exosome isolation, miRCURY RNA Isolation Kit-Cell and Plant	RT-qPCR	Diagnostic and prognostic
Barceló et al., 2020 [20]	PCa–vasectomized/PCa–non-vasectomised/BPH–non-vasectomized/healthy controls–vasectomized/healthy controls–non-vasectomized	8/16/5/5/9	miRNA	miRNA panel (diagnostic: miR-142-3p, miR-223-3p, miR-93-5p; prognostic: miR-30d-5p, miR-93-5p)	Differential centrifugation (miRCURY RNA Isolation Kit-Cell and Plant (Exiqon))	RT-qPCR	Diagnostic and prognostic
Mercadal et al., 2020 [21]	PCa-V/PCa-noV/BPH-noV/HCt-V	2/7/5/5	Exosomal miRNA	16 human miRNAs (miR-142-3p, miR-196b-3p, miR-30c-5p, miR-92a-3p, etc.)	Ultracentrifugation/miRCURY Cell/Urine/CSF (1500× *g*)/ExoGAG (3500× *g*)	RT-qPCR (LNA™), Nanoparticle tracking analysis, Flow cytometry	Diagnostic
Abramovic et al., 2021 [22]	PCa/BPH	65/58	miRNAs	miR-375-3p, miR-182-5p, miR-21-5p, miR-148a-3p	miRNeasy Serum/Plasma Advanced Kit (Qiagen)	Reverse transcription (TaqMan™ MicroRNA Reverse Transcription Kit) → ddPCR (QX200 Droplet Digital PCR System)	Diagnostic (no statistically significant differences were found) and prognostic
Lorente et al., 2021 [23]	healthy controls/vasectomized/prostate pathology (PCa/PIN/rare atypical glands)	5/5/4 (2/1/1)	Small non-coding RNAs (miRNA, tRNA, piRNA)	hsa-miR-143, hsa-miR-199a, miR-127, miR-4800, miR-187; tRNA-Gly-GGY, tRNA-Ile-ATT	Density gradient centrifugation (for cell enrichment), TRIzol-based RNA extraction, RNeasy mini kit	RT-PCR, small RNA-Seq	Diagnostic
Ferre-Giraldo, A. et al., 2024 [24]	PCa/BPH/HCt-V/HCt-noV	29/8/12/11	Small extracellular vesicle (sEV)-derived tRNA fragments (tsRNAs)	5′-M-tRNA-Gln-TTG-3-3_L30 5′-tRNA-Glu-TTC-9-1_L30 5′-tRNA-Val-CAC-3-1_L30 5′-M-tRNA-Leu-TAG-1-1_L26	Differential Centrifugation, Microfiltration and Ultracentrifugation, RNA extraction (miRNeasy Micro Kit (Qiagen))	RT-qPCR	Diagnostic and prognostic
Pelloni, M. et al., 2017 [25]	TC pre-orchiectomy (S/non-S)//TC 30 days post-orchiectomy//healthy controls	28 (23/5)//20//28	miRNAs	hsa-miR-371, hsa-miR-372, hsa-miR-373, hsa-miR-302a, hsa-miR-302b, hsa-miR-302c, hsa-miR-302d, hsa- miR-142-3p, hsa-miR-34b, hsa-miR-27a, hsa-miR-590-5p and hsa-miR-374	TRIzol-based RNA extraction (seminal plasma)/miRNeasy Serum/Plasma kit (serum)	RT-qPCR, TaqMan Array Cards A + B 3.0	Diagnostic
Radtke, A. et al., 2019 [26]	TGCT/healthy controls/men undergoing fertility testing	20/30/38	miRNAs	miR-371a-3p	miRNeasy Kit (Qiagen)	RT-qPCR (TaqMan)	Diagnostic (ineffective for cancer diagnosis, suitable for fertility determination)
Spiller, C.M. et al., 2020 [27]	TGCT//GCNIS//controls (normospermic/azoospermic)	Serum: 217//5//48; Seminal plasma: 3//1//90 (15/75)	Protein/miRNA	CRIPTO (TDGF1)/miR-371a-3p	miRNA: magnetic bead-based isolation CRIPTO: was not isolated	miRNA: RT-qPCR; CRIPTO: ELISA (enzyme immunoassay)	Diagnostic and prognostic
Mørup, N. et al., 2021 [28]	TGCTs (seminoma/non-seminoma)//GCNIS-only (germ cell neoplasia in situ)//healthy control (very heterogeneous)	18 (8/10)//5//25	Small non-coding RNAs (piRNA, miRNA, snRNA)	hsa-miR-6782-5p	Centrifugation (800 *g*, 16,000 *g*), TRIzol-based RNA extraction	small RNA sequencing, RT-qPCR (TaqMan)	Diagnostic
Dupont et al., 2023 [29]	Testicular germ cell tumors/healthy controls	16/9	Small non-coding RNAs	miR-224, piR-has-23,921, mature-tRNA-His-GTG	TRIzol-based RNA extraction	Next-Generation Sequencing	Diagnostic
Ferrara, C. et al., 2024 [30]	TGCT/fertile controls/subfertile controls	Initial analysis: 3/3/3 Validation: 8/8/8 (miR-221-3p and miR-222-3p); 6/6/6 (miR-126-3p)	miRNAs	miR-221-3p, miR-222-3p, and miR-126-3p	Qiagen miRNeasy Serum/Plasma Kit	Initial analysis: RT-qPCR Validation: dPCR	Diagnostic
**Circulating tumor cells**							
Rzhevskiy, A.S. et al., 2022 [31]	PCa/healthy controls	15/15	CTC	CTC number	Inertial microfluidics	Immunofluorescence Staining (CK, PSMA, GPC-1, DAPI), Laser-scanning confocal microscope	Diagnostic and prognostic
Saitta, C. et al., 2023 [32]	Localized PCa (patients were stratified in detail according to the Gleason scale, ISUP, TNM stage, etc.)	356 agreed to donate seminal fluid, 100 donated seminal fluid and urine	CTC	CTC number	FACS Sorting	FACS Sorting	Diagnostic (insignificant)
**Protein Biomarkers**							
Tajiri, M. et al. 2008 [33]	PCa/commercially available specimen	2/nm	Proteins	The presence of α2.3-linked sialic acids on PSA glycans	Sulfate-polyacrylamide gel electrophoresis (SDS PAGE), In-gel de-sialylation, In-gel AE & LEP digestion, Reversed-phase LC	Mass spectrometry (MALDI-TOF MS and MALDI-IMS/MS)	Diagnostic
van Casteren, N.J. et al., 2008 [34]	Suspicion of TGCT (Seminoma/Nonseminoma/Combined tumor/CIS with burned-out tumor/Non-germinogenic testicular tumors) //Postorchidectomy (Nonseminoma/Combined tumor)//Bilateral testicular microlithiasis (CIS/no malignancy)//healthy controls	14 (4/4/ 2/2/2)//14 (11/3)//13 (3/10)//15	Protein	OCT3/4 (POU5F1)	Cytocentrifugation (Cytospin)	Immunohistochemistry	Diagnostic
Zenzmaier, C. et al., 2011 [35]	PCa/nonPCa	The main cohort: 40/41 A subgroup with long-term follow-up: 21/25	Protein	Dkk-3 (Dickkopf-related protein 3)	Centrifugation (Percoll)	IEMA (Indirect Immunoenzymometric Assay)	Diagnostic
Nielsen, J.E. et al., 2012 [36]	TGCT (seminoma)/TGCT (non-seminoma)/CIS	12/8/2	Proteins	AP-2c (TFAP2C), NANOG, OCT3/4, KIT, placental-like alkaline phosphatase (PLAP), M2A/PDPN and MAGE-A4	Cytocentrifugation (Cytospin)	Immunohistochemistry and histochemistry	Diagnostic
Saraon, P. et al., 2012 [37]	PCa High-grade (Gleason ≥ 7)/PCa Low-grade (Gleason ≤ 6)/prostatitis/negative biopsy	13/8/8/8	Protein	PROS1	Centrifugation, standard proteomic steps: dialysis, lyophilization, denaturation, reduction, alkylation, and enzymatic hydrolysis with trypsin.	ELISA for seminal plasma; Immunohistochemistry for tissue	Diagnostic and prognostic
Neuhaus, J. et al., 2013 [38]	PCa/BPH/chronic prostatitis/healthy controls	70/21/25/9	Peptides	Fragments of N-acetyllactosaminide beta-1,3-N-acetylglucosaminyl-transferase, prostatic acid phosphatase, stabilin-2, GTPase IMAP family member 6, semenogelin-1/2, etc.	Centrifugation, top-down approach	Capillary electrophoresis mass spectrometry and nanoflow liquid chromatography-tandem MS (nanoLC-MS/MS).	Diagnostic and prognostic
Hoei-Hansen, C.E. et al., 2007 [39]	TGCT before orchidectomy//TGCT after orchidectomy//Carcinoma in situ-only//Other testicular tumors/lesions//Infertility//other control participants	65//42//6//8//294//88	Proteins	AP-2γ (TFAP2C) (Additionally, OCT-3/4, NANOG, and PLAP were tested)	Cytocentrifugation (Cytospin)	Immunocytochemistry	Diagnostic
Satie, A-P. et al., 2009 [40]	TGCT/healthy controls	57/65	Proteins	MAGE-A4 and NY-ESO-1	N/A	Immunocytochemistry	Diagnostic
Lippert, S. et al., 2015 [41]	BPH/localized PCa/metastatic PCa/healthy controls	12/34/35/120	Peptide/protein and mRNA	C-type natriuretic peptide (CNP), proCNP and NPR-B (CNP receptor)	NucleoSpin RNA kit for mRNA isolation; trypsin treatment and centrifugation for protein isolation	qPCR for mRNA and Radioimmunological assays for proteins	Diagnostic and prognostic
Karakosta, T.D. et al., 2016 [42]	PCa/negative biopsy controls/healthy controls	Seminal plasma: 21/20/10 Blood serum: 36/22/3	Proteins	15 kallikrein-related peptidases (KLK4 is the main focus of the research)	Centrifugation, protein denaturation, disulfide bond reduction, alkylation, enzymatic hydrolysis (digestion), reverse-phase sorption, and immuno-enrichment	Mass spectrometry (SRM/PRM) and ELISA	Diagnostic—ineffective
Etheridge, T. et al., 2018 [43]	PCa/healthy controls	28/15	Protein	AMACR	Centrifugation	Indirect sandwich ELISA, chemiluminescence assay	Diagnostic
Drabovich, A.P. et al., 2019 [44]	PCa (low-grade/intermediate-grade/high-grade)//nonPCa	152 (94/38/20)//67	Proteins	TGM4 and other 18 proteins (PAEP, CD9, COR1B, KLK3, TMPRSS2, etc.)	Standard proteomic methods (denaturation, reduction, alkylation and enzymatic hydrolysis by trypsin)	Selected reaction monitoring, ELISA	Diagnostic and prognostic
Ruiz-Plazas, X. et al., 2019 [45]	PCa low-risk (ISUP grade I-II) and high-risk (ISUP grade III-V)	98 (63.3%:36.7%)	Proteins and mRNA	Proteins: sTWEAK, sFn14, sCD163, sCXCL5, sCCL7; mRNA: Fn14, CD163, KLK2, KLK3, CXCR2, CCR3	Centrifugation, TRIzol based RNA extraction	ELISA, qRT-PCR	Prognostic
**Metabolic Biomarkers**							
Gregório, E.P. et al., 2019 [46]	PCa/BPH	31/28	Metabolite	Citrate concentration	Centrifugation and lyophilization	1HNMRS—High-resolution nuclear magnetic resonance spectroscopy	Diagnostic
Barrio-Muñoz, M. et al., 2015 [47]	PCa/nonPCa	22/21	Reactive oxygen species	Total level of ROS	Centrifugation	Nitro blue tetrazolium assay (NBT) and spectrophotometry	Diagnostic (insignificant)
Lakpour, N. et al., 2024 [48]	TC/healthy controls	9/10	Metabolites	phenylalanine, tyrosine, lipids, proteins, phenols etc.	Methanol and H2O (2:1) → centrifugation	Raman spectroscopy	Diagnostic

## Data Availability

No new data were created or analyzed in this study.

## Supplementary Materials

The following supporting information can be downloaded at: https://www.mdpi.com/article/10.3390/biomedicines14050966/s1, Figure S1: QUADAS-2 Risk of Bias assessment; Table S1: PRISMA checklist; Table S2: Evaluation of the level of evidence according to Oxford Centre for Evidence-Based Medicine.

## Abbreviations

The following abbreviations are used in this manuscript:

AUC	Area Under the Curve
BPH	Benign Prostatic Hyperplasia
CIS	Carcinoma In Situ
csPCa	Clinically Significant Prostate Cancer
CTC	Circulating Tumor Cell
ctDNA	Circulating Tumor DNA
cfDNA	Cell-Free DNA
EV	Extracellular Vesicle
miRNA (miR)	MicroRNA
mRNA	Messenger RNA
PCa	Prostate Cancer
PSA	Prostate-Specific Antigen
ROS	Reactive Oxygen Species
RT-qPCR	Reverse Transcription Quantitative Polymerase Chain Reaction
SF	Seminal Fluid
sncRNA	Small Non-Coding RNA
TC	Testicular Cancer
TGCT	Testicular Germ Cell Tumor
tsRNA	tRNA-Derived Small RNA
GCNIS	Germ Cell Neoplasia In Situ
